# The effect of subjective understanding on patients’ trust in AI pharmacy intravenous admixture services

**DOI:** 10.3389/fpsyg.2024.1437915

**Published:** 2024-09-05

**Authors:** Yongzhi Gong, Xiaofei Tang, Haoyu Peng

**Affiliations:** ^1^School of Business Administration, Southwestern University of Finance and Economics, Chengdu, China; ^2^Graduate Institute of Science, University of Peradeniya, Peradeniya, Sri Lanka

**Keywords:** artificial intelligence, intravenous admixture service, subjective understanding, informed consent, trust

## Abstract

**Introduction:**

Medical services are getting automated and intelligent. An emerging medical service is the AI pharmacy intravenous admixture service (PIVAS) that prepares infusions through robots. However, patients may distrust these robots. Therefore, this study aims to investigate the psychological mechanism of patients’ trust in AI PIVAS.

**Methods:**

We conducted one field study and four experimental studies to test our hypotheses. Study 1 and 2 investigated patients’ trust of AI PIVAS. Study 3 and 4 examined the effect of subjective understanding on trust in AI PIVAS. Study 5 examined the moderating effect of informed consent.

**Results:**

The results indicated that patients’ reluctance to trust AI PIVAS (Studies 1–2) stems from their lack of subjective understanding (Study 3). Particularly, patients have an illusion of understanding humans and difficulty in understanding AI (Study 4). In addition, informed consent emerges as a moderating factor, which improves patients’ subjective understanding of AI PIVAS, thereby increasing their trust (Study 5).

**Discussion:**

The study contributes to the literature on algorithm aversion and cognitive psychology by providing insights into the mechanisms and boundary conditions of trust in the context of AI PIVAS. Findings suggest that medical service providers should explain the criteria or process to improve patients’ subjective understanding of medical AI, thus increasing the trust in algorithm-based services.

## Introduction

Medical services are getting automated and intelligent. Because of the ability to quickly process large amounts of medical information and provide users with structured results, artificial intelligence (AI) is increasingly assisting human physicians in medical diagnosis (Cestonaro et al., 2023; Cadario et al., 2021; Juravle et al., 2020), or even retrieving and dispensing pills for patients based on prescriptions (Starke et al., 2021; Leo and Huh, 2020). AI pharmacy intravenous admixture service (PIVAS) is an emerging medical service that prepares the fluid drugs and nutrients for infusion through AI robots. There is evidence that an increasing number of hospitals are using AI robots for intravenous medication preparation (Yang et al., 2023; Nurgat et al., 2021; He et al., 2014), such as RIVA robot. These AI robots can independently replace human pharmacists in medication dispensing (He et al., 2014). However, with the popularity of AI PIVAS, concerns and controversies have arisen. The public may be suspicious of or distrust these AI robots (Durán and Jongsma, 2021). But to date, few studies have shown how trust in AI PIVAS is affected when PIVAS is administered by an AI robot rather than a pharmacist.

Previous studies have explored the factors affecting AI trust. On one hand, trust in AI can be influenced by several human factors such as AI anxiety (Kaya et al., 2024), religion (Jackson et al., 2023), and beliefs about AI (Xie et al., 2024; Zhang et al., 2023), etc. On the other hand, the impact of AI attributes on trust is prominent, including anthropomorphism (Bergner et al., 2023; Huang and Wang, 2023), usefulness and reliability (Ismatullaev and Kim, 2024; Choung et al., 2023), and types of AI (Clegg et al., 2024; Usman et al., 2024). Most importantly, transparency is considered to be the bridge that builds user trust (Wang and Ding, 2024). Increasing the transparency, usability, and security of AI is an important factor in building trust (Wang and Siau, 2019). Based on transparency, we argue that an important cause of trust barriers related to AI PIVAS is that AI is perceived to be opaque, that is, AI is a “black box.” Patients’ inability to subjectively understand how AI performs PIVAS undermines their trust in AI PIVAS.

Subjective understanding can be interpreted as people’s subjective knowledge, or what they think they know (Cadario et al., 2021). The algorithmic characteristics of AI dictate opacity and inexplicability, making it hard for people to understand its principles subjectively, leading to difficulty in understanding AI. Instead, the subjective understanding of human decision-making stems from a belief that “introspection” provides direct access to the mental processes by which people make decisions (Nisbett and Wilson, 1977). However, people do not actually have access to their own associative mechanisms (Morewedge and Kahneman, 2010). In essence, the assessments made by humans are often as much of a “black box” as the decisions made by AI. Nevertheless, people often claim to understand humans better than AI, which is an illusion of understanding. They believe they have a good understanding of something, but in reality, their comprehension is not as strong as they assume (Cadario et al., 2021; Bonezzi et al., 2022). Therefore, we argue that subjective understanding is an important role for trusting AI PIVAS, and that illusion of understanding humans and difficulty in understanding AI are the drivers. We also examined a boundary condition—that is, whether the hospital informed the patients of the PIVAS. Our aim was to demonstrate that patients have a stronger subjective understanding and higher trust in an explainable AI PIVAS.

## Theoretical background and hypotheses

### Trust in AI PIVAS

Trust is a subjective judgment formed by the trustor, based on their perception of the trustee’s characteristics and their past experiences with them (Li et al., 2024). Understanding the dynamics of trust between AI and humans is crucial, especially in the life-and-death healthcare field (Asan et al., 2020). When patients trust healthcare agents, they actively engage in healthcare and are satisfied with healthcare services (Wu et al., 2016), thus demonstrating the importance of trust in AI healthcare. However, people by default tend to trust AI less than humans (Williams and Lim, 2024) and do not increase their trust in AI even when they know that the algorithm is superior (Juravle et al., 2020). Studies have found that people are reluctant to trust AI technology in the medical field. For example, participants preferred medical services provided by human physicians over those provided by AI, even though the AI performed as well or better than human physicians (Longoni et al., 2019), because they think AI will ignore their unique characteristics. Studies have also shown that people are more averse to AI making medical decisions than human doctors, regardless of the outcome (Bigman and Gray, 2018). Therefore, we propose that:

H1: Patients trust in AI PIVAS less than in human PIVAS.

### Subjective understanding

Subjective understanding is mental cognition based on people’s subjective knowledge (Cadario et al., 2021). Research has found that trust in a new technology depends not only on past experience but also on understanding of the technology. Understanding generates trust, which is more stable than trust based only on performance reliability (Lee and See, 2004). Thus, when the algorithms are comprehensible, trust is likely to be enhanced. However, with the development of AI, algorithms have become a “black-box,” making it difficult for users to understand their decision-making process (Subramanian et al., 2024; Raees et al., 2024). Essentially, the problem of black-box algorithms is one of transparency. Transparency reflects whether the basic operating rules and internal logic of the technology are obvious to users and is considered crucial for trusting new technologies (Li et al., 2024). Due to the lack of transparency in AI, people are unable to understand why AI produces a particular output and make decisions accordingly (Schlicker et al., 2021). It is difficult for patients to determine whether they can trust AI medical advice and make decisions accordingly. This could hinder trust in medical AI and even lead to paralysis of the medical decision making (Triberti et al., 2020). Therefore, people’s limited understanding of how algorithms work is an important reason for distrust in AI (Yeomans et al., 2019). Thus, we propose that:

H2: Subjective understanding mediates the relationship between AI PIVAS and trust.

### Illusion of understanding vs. difficulty in understanding

Although people think they can understand human decisions, this is often not the case. Sometimes, specialists are unable to provide an explanation, such as when a doctor makes a diagnosis without explaining to the patient how it was made (Mangano et al., 2015). As a result, human decision makers may also be opaque and just like a black box as AI. However, people are more likely to trust humans than AI. We argue that people have an illusion of understanding humans, believing that they know more about humans than algorithms. However, they do not understand either AI or humans. The essence of the illusion of understanding is that people often overestimate how well they understand how things work, a phenomenon known as the illusion of explanatory depth (Rozenblit and Keil, 2002). People perceive what others think and then develop feelings in certain situations by projecting their own thoughts, feelings, and preferences onto others (Bonezzi et al., 2022). The more similar the projected person is to oneself, the greater the degree of this projection. As perceived similarity decreases, the degree to which people project onto others decreases (Ames, 2004). Since people are more similar to other humans than to algorithms (Gray et al., 2007), they are more likely to project their intuitive understanding of the decision process onto humans, thus relying on their understanding of the decision process to perceive how other humans make decisions. This is misleading and leads to an illusion of understanding. In contrast, people have difficulty in understanding AI. Therefore, we propose that:

H3: Patients have higher trust in human (vs. AI) PIVAS due to their illusion of understanding humans (vs. difficulty in understanding AI).

### Informed consent

The lack of transparency in AI makes it difficult for people to understand its decision-making process. However, there are two sources to enhance transparency: an explanation of how the algorithm works, and a reflection of AI reliability (Subramanian et al., 2024). As such, explainability has been identified as a key factor in the adoption of AI (Hamm et al., 2023). In medical services, informed consent is a common service with an explanatory nature, which provides sufficient information about a treatment or intervention (Wałdoch, 2024). AI PIVAS is an emerging medical service and has not yet been generally accepted by the public. Without informed consent, there is a high risk of medical disputes once the AI fails (Wałdoch, 2024). For example, in 2013, a hospital in Nanjing, China, failed to fully and objectively inform patients about a new medical technology that was still in the promotion stage, resulting in the patient suing the hospital for financial compensation. Essentially, informed consent is an explanation mechanism that enables patients to better understand the relevant medical situation (Wałdoch, 2024). Since an explainable AI can be fully understood (Gunning et al., 2019), patient’s subjective understanding of AI PIVAS should be enhanced by informed consent (Zhang et al., 2024). In summary, we propose that:

H4: Informed consent moderates the relationship between AI PIVAS and subjective understanding. Specifically, patients have higher subjective understanding of AI PIVAS with informed consent.

**Figure 1:**
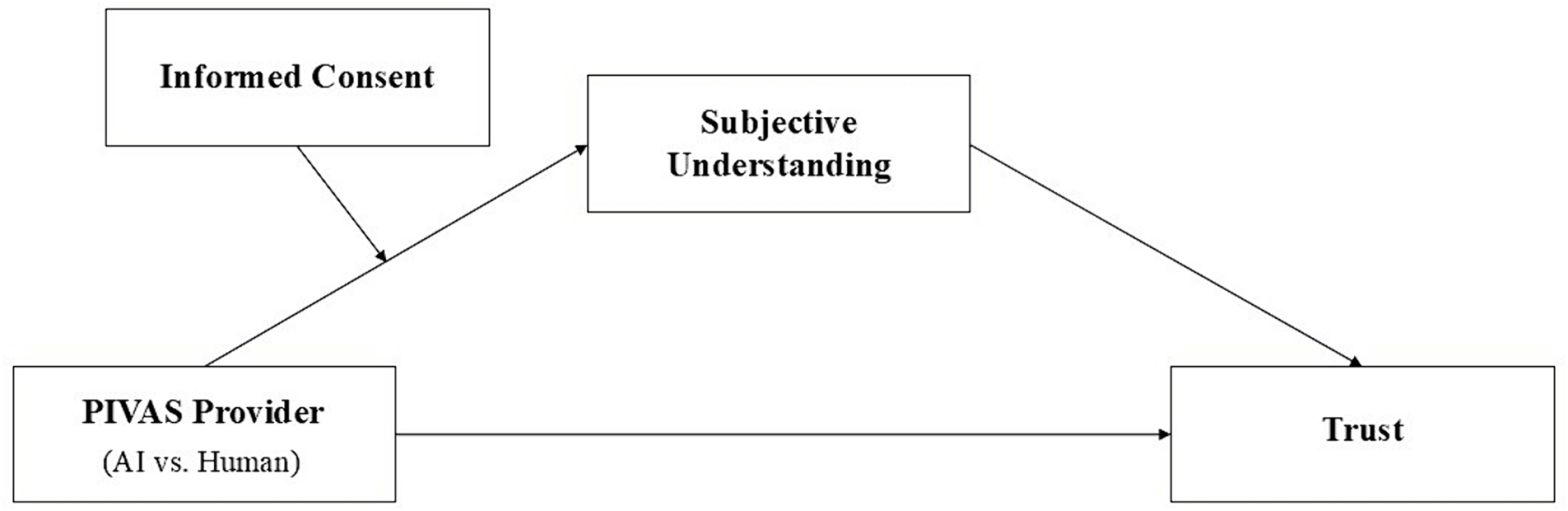
Conceptual model.

Overall, the conceptual model of our study is as shown in Figure 1.

### Overview of studies

We explored the impact of subjective understanding on patients’ trust in AI PIVAS through a field study (Study 1) and four experimental studies (Study 2–5). All reported studies were conducted in full accordance with the Declaration of Helsinki and included obtaining informed consent from all participants before they were able to take part in the study. We chose four common diseases requiring PIVAS as experimental scenarios: rhinovirus infection, wound infection, diarrhea, and acute gastritis.

Study 1–2 verified the assumption that patients are more likely to trust human PIVAS than AI PIVAS, testing the main effect. Study 3 tested the mediating role of subjective understanding between AI PIVAS and trust, and excluded other alternative explanations (i.e., objective understanding). Study 4 further explored the role of patients’ illusion of understanding humans and the role of difficulty in understanding AI. Study 5 considered other confounds and examined the moderating effect of informed consent on the relationship between AI PIVAS and subjective understanding.

## Study 1

Study 1 investigated whether patients are less likely to pursue PIVAS administered by AI vs. human providers. We explored this question in a real-world setting, offering patients the opportunity to choose the PIVAS provider for the intravenous therapy.

### Participants and procedure

We conducted a field study of 200 patients (*M*_age_ = 29.86, *SD* = 7.05, 60.5% female) undergoing intravenous therapy over 10 days at a large public hospital in western China, which provides AI PIVAS of all-species. We told patients that we were conducting a survey on intravenous infusion. Patients were told that the hospital would provide two types of PIVAS to preparing their infusion medications, one by pharmacists and the other by AI robots. We also informed patients that they would not interact with the PIVAS provider that neither PIVAS incurred costs, and that there was no significant difference in performance between the two. Then, patients were asked to choose between pharmacists and AI robots. In this case, patients’ choice of PIVAS provider was not related to their preferences for interaction, nor was it related to perceived costs and performance of PIVAS.

Next, we had patients report their trust in both pharmacists (*α* = 0.80) and AI robots (*α* = 0.85) on a seven-point scale in the following four items (Anderson and Dedrick, 1990): “I trust pharmacists (robots) so much that I will always accept the medication they prepare for me,” “I trust the judgment of pharmacists (robots) in the preparation of my medication,” “I trust pharmacists (robots) to put my medical needs above all other considerations when preparing medication,” and “I trust pharmacists (robots) to tell me if they have made a mistake in preparing my medication.” Finally, we counted the gender and age of the patients and provided them with gifts of appreciation.

### Results and discussion

We counted the proportion of patients choosing a PIVAS provider (pharmacists vs. AI robots). Among the 200 patients, 187 patients (93.5%) chose pharmacists and only 13 patients (6.5%) chose AI robots. Further analysis showed that after controlling for age and gender, patients’ trust in pharmacists was significantly higher than in AI robots [*M*_pharmacists_ = 5.07, *SD* = 0.95; *M*_AI_ = 3.95, *SD* = 0.93; *t* (199) = 11.91, *p* < 0.001, *d* = 1.19].

The results of Study 1 provide preliminary evidence that patients are reluctant to use AI PIVAS compared to human PIVAS. Faced with a choice between pharmacists and AI robots, patients preferred the pharmacists to prepare their infusion medications, even if both performed equally well. This may be caused by patients generally having a higher level of trust in pharmacists than AI robots. However, in real-world situations, it is possible that patients’ choices may be confounded by other factors, leading to biased conclusions. Therefore, in the following studies, we used cleaner scenario experiments to further test our hypotheses.

## Study 2

Study 2 was designed to verify that patients have higher trust in human PIVAS than in AI PIVAS. In Study 2, we used rhinovirus infection as a scenario for PIVAS.

### Participants and procedure

We recruited 140 valid participants (*M*_age_ = 29.07, *SD* = 7.85, 57.9% female) on Credamo to participate in the study in exchange for monetary compensation. We adopted a between-group factorial design (PIVAS provider: AI vs. human). Participants were randomly assigned to one of these two conditions and read the following experimental material (see Appendix 1):

“*Imagine that you have recently developed a fever and feel ill because you have a rhinovirus infection. You decide to go to a well-known hospital for treatment. After diagnosis, the doctor determines that your condition is more serious and that you need an infusion to recover better, and prescribes one. You followed the doctor’s advice and are ready to receive the infusion*.”

In the human condition, participants were told that “*the hospital provides Pharmacy Intravenous Admixture Services that all medications are prepared by pharmacists*.” In the AI condition, participants were told that “*the hospital provides Pharmacy Intravenous Admixture Services that all medications are prepared by AI robots*.” Subsequently, participants in the AI (human) condition viewed a picture of an AI PIVAS (a human PIVAS) to reinforce the experimental scenario (see Appendix 2).

Then participants were asked to report their trust (*α* = 0.83) in the PIVAS provider as in Study 1. As an attention check, participants were asked to indicate whether they remembered who prepared the medication in the scenario (pharmacists, AI robots, or no memory). Finally, participants provided generic demographic information such as gender, age, and education. We used the same attention check and demographic questions in subsequent studies.

### Results and discussion

The results showed that after controlling for demographics, participants’ trust was significantly higher in the human condition (*M* = 5.76, *SD* = 0.81) than in the AI condition [*M* = 4.79, *SD* = 1.24; *t*(138) = 5.47, *p* < 0.001, *d* = 0.93]. The results of Study 2 showed that patients had a stronger tendency to trust human PIVAS than AI PIVAS. This is consistent with the findings of previous studies on algorithm aversion. Study 3 introduced subjective understanding as a mediator in an attempt to clarify the mechanisms by which patients develop differences in trust between AI PIVAS and human PIVAS.

## Study 3

Study 3 aimed to verify that the reason patients prefer to trust human PIVAS over AI PIVAS is that they subjectively do not understand AI PIVAS. In Study 3, we used wound infection as a scenario for PIVAS.

### Participants and procedure

We recruited 130 valid participants (*M*_age_ = 29.69, *SD* = 7.34, 62.3% female) on Credamo to participate in the study in exchange for monetary compensation. We adopted a between-group factorial design (PIVAS provider: AI vs. human). Participants were randomly assigned to one of these two conditions and read the experimental materials, as in Study 2 (see Appendix 1). The only difference was that participants were told they felt sick because of an infection caused by a fall and subsequent injury. Afterwards, participants reported their trust (*α* = 0.88) and subjective understanding (*α* = 0.85). The measure of subjective understanding was adapted from that of subjective knowledge (Cadario et al., 2021): “To what extent do you understand the information based on which the pharmacists (AI robots) are preparing the medication?” “To what extent do you understand the process of preparing medication by the pharmacists (AI robots)?” and “To what extent do you understand the efficacy of the pharmacists (AI robots) preparing the medication?” (1: completely do not understand; 7: completely understand).

In addition, Study 3 was designed to rule out the alternative explanation that patients exhibit different levels of trust in human PIVAS and AI PIVAS due to differences in objective understanding. We therefore measured participants’ objective knowledge of PIVAS. We consulted with the medical professionals of a hospital and finalized three objective differences between AI PIVAS and human PIVAS. First, the human PIVAS requires pharmacists to check the prescription information once, while AI PIVAS requires both pharmacists and robots to check the prescription information twice in total. Second, in human PIVAS, pharmacists can tilt and pull the syringe, while in AI PIVAS, the syringe is fixed vertically. Third, the residual rate (the ratio of the residual drug solution to the overall mixed drug solution) of human PIVAS is usually about 5%, while the residual rate of AI PIVAS is around 1%. Based on these three differences, we created three multiple-choice questions. Each question had a correct option for human PIVAS, a correct option for AI PIVAS, and an incorrect option (see Appendix 4). For example, “How many times does the pharmacists (AI robots) need to check the prescription during the process of medication?” There were three options: one time (correct answer for human PIVAS), two times (correct answer for AI PIVAS), and three times (incorrect answer for both human PIVAS and AI PIVAS). We obtained the objective understanding of the participants by summarizing the correct answers, so the scores ranged from 0 to 3. Finally, the participants completed the attention check and demographic questions.

### Results

#### Main effect analysis

The results showed that after controlling for demographics, participants’ trust was significantly higher in the human condition (*M* = 5.75, *SD* = 0.73) than in the AI condition [*M* = 4.78, *SD* = 1.52; *t* (128) = 4.63, *p* < 0.001, *d* = 0.81]. In addition, participants’ subjective understanding of human PIVAS (*M* = 5.24, *SD* = 0.97) was significantly higher than that of AI PIVAS [*M* = 4.41, *SD* = 1.30; *t* (128) = 4.13, *p* < 0.001, *d* = 0.72]. However, participants’ objective understanding of human PIVAS (*M* = 1.14, *SD* = 0.66) and AI PIVAS (*M* = 0.95, *SD* = 0.76) did not differ significantly [*t* (128) = 1.48, *p* > 0.05, *d* = 0.26]. The results suggest that patients are willing to trust human PIVAS because they subjectively understand pharmacists more than they understand AI. But objectively, they understand neither pharmacists nor AI. Next, we tested the mediating role of subjective understanding.

#### Mediating effect analysis

**Figure 2:**
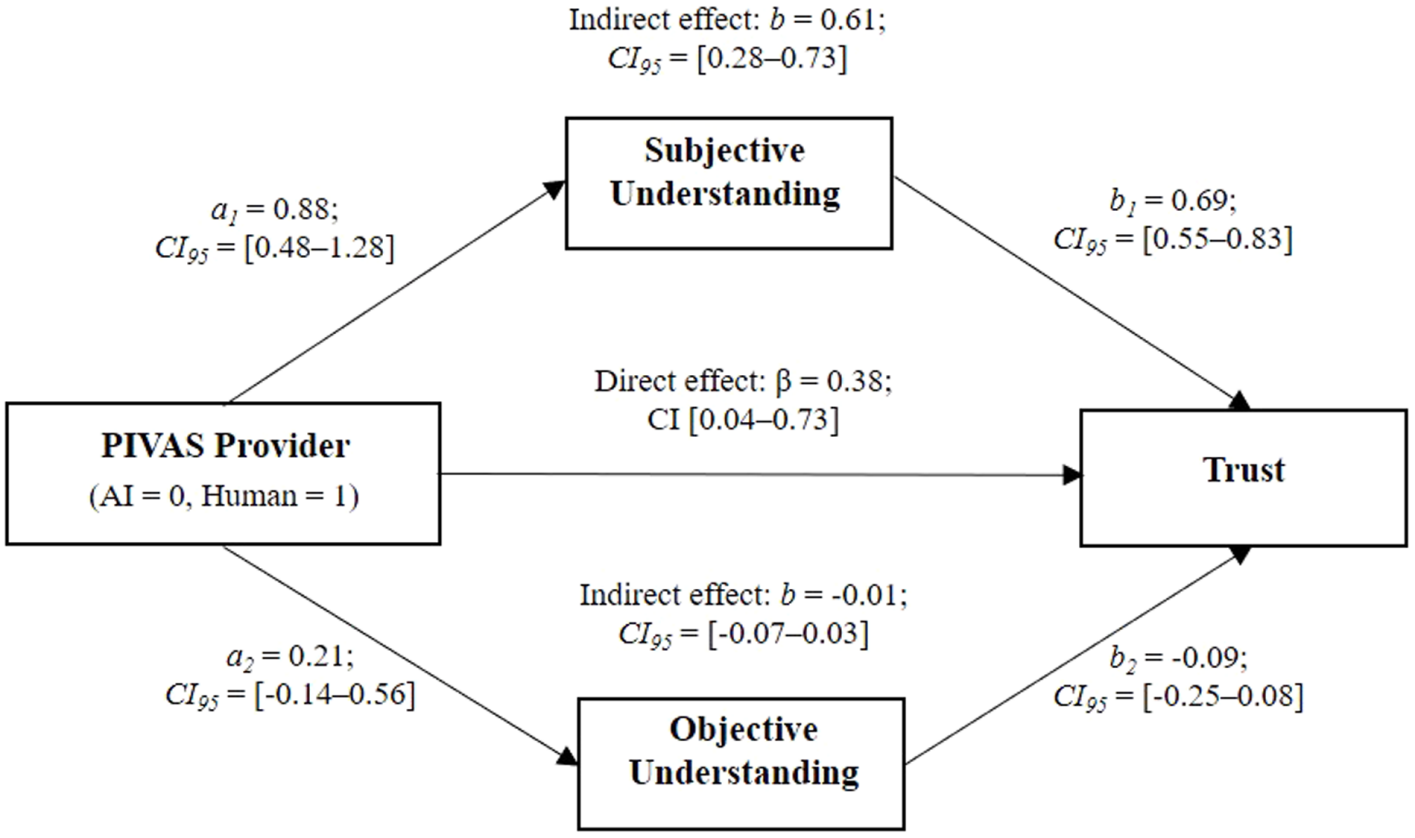
The mediation effect of subjective understanding.

We used Bootstrapping (PROCESS Model 4) to analyze the mediating role of subjective understanding. We first transformed participants’ scores of objective understanding into *Z* scores and then coded the independent variable as 0 (AI PIVAS) and 1 (human PIVAS). The results (see Figure 2) showed a significant direct effect of PIVAS provider (*b* = 0.38, *CI*_95_ = [0.04, 0.73]). The indirect effect of subjective understanding was significant (*b* = 0.61, *CI*_95_ = [0.28, 0.73]), and the direction of the effect confirmed that patients had a higher subjective understanding of human PIVAS (compared to that of AI PIVAS), which triggered stronger trust. The indirect effect of objective understanding was insignificant (*b* = −0.01, *CI*_95_ = [−0.07, 0.03]), indicating that this variable could not explain the observed differences in trust.

### Discussion

Study 3 further verified the main effect of PIVAS provider on trust again and clarified the mechanism. This effect of PIVAS provider on trust is formed through subjective understanding. Notably, Study 3 showed an equivalent level of objective understanding between humans and AI, yet a significant disparity exists in their subjective understanding. To further explore the mediating mechanism of subjective understanding, Study 4 examined the role of illusion of understanding and difficulty in understanding.

## Study 4

Study 4 aimed to further explore the mediating mechanism of subjective understanding. We argue that patients are more willing to trust human PIVAS than AI PIVAS due to the illusion of understanding humans and difficulty in understanding AI. Thus, if the medication is prepared by pharmacists, then having participants explain the process should reduce the subjective understanding of human PIVAS. In Study 4, we used diarrhea as a scenario for PIVAS.

### Participants and procedure

Study 4 was a 2 (PIVAS provider: AI vs. human) × 2 (rating order: pre-explanation vs. post-explanation) mixed design. We recruited 130 valid participants (*M*_age_ = 28.65, *SD* = 7.25, 60.8% female) on Credamo to participate in the study in exchange for monetary compensation. Participants were randomly assigned to one of the two PIVAS conditions (between-group factor) and they were told to report their first subjective understanding before the explanation and the second subjective understanding after the explanation (within-group factor). Participants first read the experimental materials (see Appendix 1). They were told to imagine that they were suffering from diarrhea and feeling ill because they had eaten spoiled food.

Then participants made the first rating of subjective understanding of PIVAS (*α* = 0.92). To measure the degree of illusion on the first rating, we drew on similar interventions in the psychology and knowledge domains (Bonezzi et al., 2022; Vaupotič et al., 2022). Participants were asked to give an explanation of the process of medication preparation and to describe in as much detail as possible the specific process of PIVAS (see Appendix 3). After completing the explanation, participants provided a second rating of their subjective understanding of PIVAS (*α* = 0.82). Finally, the participants completed the attention check and demographic questions.

### Results

ANOVA analysis showed a significant main effect of PIVAS provider [*F* (1,129) = 165.91, *p* < 0.001, η^2^ = 0.57] after controlling for demographics. Participants’ subjective understanding of human PIVAS was significantly higher than that of AI PIVAS in both the pre-explanation condition [*M*_human_ = 5.65, *SD* = 0.11; *M*_AI_ = 3.30, *SD* = 0.11; *F* (1,129) = 224.93, *p* < 0.001, η^2^ = 0.64] and the post-explanation condition [*M*_human_ = 4.51, *SD* = 0.10; *M*_AI_ = 3.27, *SD* = 0.10, *F* (1,129) = 77.29, *p* < 0.001, η^2^ = 0.38]. Similarly, the main effect of rating order was significant [*F* (1,129) = 5.19, *p* < 0.05, η^2^ = 0.04]. Participants’ subjective understanding was higher in the pre-explanation condition (*M* = 4.48, *SD* = 1.48) and lower in the post-explanation condition (*M* = 3.89, *SD* = 1.03). Importantly, there was a significant interaction effect [*F* (1,129) = 111.53, *p* < 0.001, η^2^ = 0.47].

**Figure 3:**
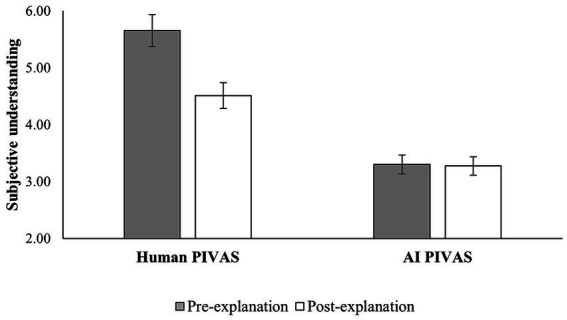
Illusion of understanding human vs. difficulty in understanding AI.

In the human group, participants’ subjective understanding was significantly higher in the pre-explanation condition (*M* = 5.65, *SD* = 0.11) than in the post-explanation condition [*M* = 4.51, *SD* = 0.10, *ΔM* = 1.14, *F* (1,129) = 234.88, *p* < 0.001, η^2^ = 0.65]. The significant decrease in participants’ subjective understanding of human PIVAS from pre-explanation to post-explanation suggests an illusion of understanding human PIVAS. However, in the AI group, participants’ subjective understanding did not differ significantly [*ΔM* = 0.03, *F* (1,129) = 0.13, *p* > 0.05, η^2^ = 0.001] in the pre-explanation condition (*M* = 3.30, *SD* = 0.11) or the post-explanation condition (*M* = 3.27, *SD* = 0.10), as shown in Figure 3. This suggests that patients have difficulty in understanding AI PIVAS.

### Discussion

Study 4 provided evidence to further explore the mediating role of subjective understanding. The findings suggested that patients may prefer to trust human PIVAS over AI PIVAS not only because they have difficulty in understanding AI, but also because they have an illusion of understanding pharmacists. Patients do not understand pharmacists as well as they think they do, and due to projection effects, their subjective understanding of pharmacists is in fact an illusion. In summary, Study 3 and 4 provided evidences that subjective understanding plays an important role in the relationship between AI PIVAS and trust. Next, Study 5 examined the moderating effect of informed consent on the relationship between AI PIVAS and subjective understanding.

## Study 5

Study 5 aimed to test whether patients’ trust in AI PIVAS would be enhanced by improving their subjective understanding. Therefore, we used informed consent as a medical service to improve patients’ subjective understanding by explaining to them the process of AI PIVAS. In addition, considering that patients’ trust may be influenced by other factors, such as attitudes toward AI (Grassini, 2023), previous experience with AI (Hu, 2021), and AI literacy (Shen and Cui, 2024), we included them as controls in the overall model. In Study 5, we used acute gastritis as the scenario for PIVAS.

### Participants and procedure

Study 5 adopted a 2 (PIVAS provider: AI vs. human) × 2 (informed consent: informed vs. controlled) between-group design. We recruited 200 valid participants (*M*_age_ = 33.74, *SD* = 12.93, 55% female) on Credamo to participate in the study in exchange for monetary compensation. Participants were randomly assigned to one of these four conditions. Participants first read the experimental material as in the previous studies (see Appendix 1). They were told that they felt sick because they had recently suffered from acute gastritis. Afterwards, participants in the informed condition read that they had received an informed consent form from the hospital after the diagnosis (see Appendix 5). In contrast, participants in the controlled condition did not read the relevant material. Then participants reported their subjective understanding (*α* = 0.92), trust (*α* = 0.90), attitudes toward AI (*α* = 0.94), previous experience with AI, and AI literacy (*α* = 0.84). Four items from Grassini (2023) were used to measure attitudes toward AI, including “I believe that AI will improve my life.” We use one item adapted from Hu (2021) to measure previous experience with AI, including “I use AI a lot in my daily life and work.” Drawing on the work of Shen and Cui (2024), we measured AI literacy on the usage dimension, including three items such as “I can skillfully use AI applications or products to help me with my daily work.” Finally, the participants completed the attention check and demographic questions.

### Results

#### Trust

Consistent with the previous studies, one-factor ANOVA results revealed that after controlling for other factors, participants’ trust in human PIVAS (*M* = 5.68, *SD* = 0.75) was higher than that in AI PIVAS [*M* = 4.36, *SD* = 1.40; *F* (1,199) = 57.34, *p* < 0.001, η^2^ = 0.23].

#### Subjective understanding

**Figure 4:**
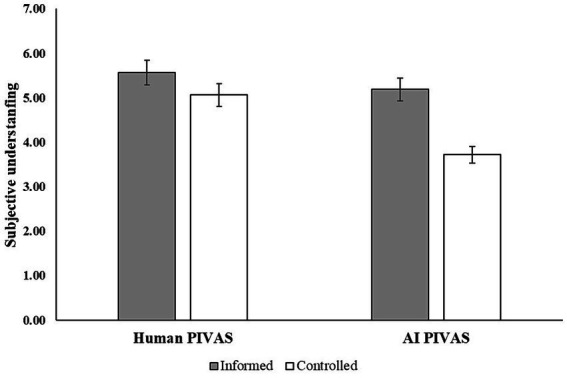
Interaction effect between PIVAS provider and informed consent.

One-factor ANOVA results (see Figure 4) revealed that after controlling for other factors, there was an interaction effect between PIVAS provider and informed consent on subjective understanding [*F* (1, 199) = 7.74, *p* < 0.001, η^2^ = 0.03]. Participants had higher subjective understanding in AI PIVAS with informed consent (*M* = 5.19, *SE* = 0.18) than without informed consent [*M* = 3.72, *SE* = 0.19, *F* (1,191) = 34.39, *p* < 0.001, η^2^ = 0.15]. However, participants’ subjective understanding in human PIVAS did not differ significantly with informed consent (*M* = 5.56, *SE* = 0.18) and without informed consent [*M* = 5.06, *SE* = 0.20, *F* (1,191) = 3.72, *p* > 0.05, η^2^ = 0.02], supporting H4.

#### Moderated mediation analysis

**Figure 5:**
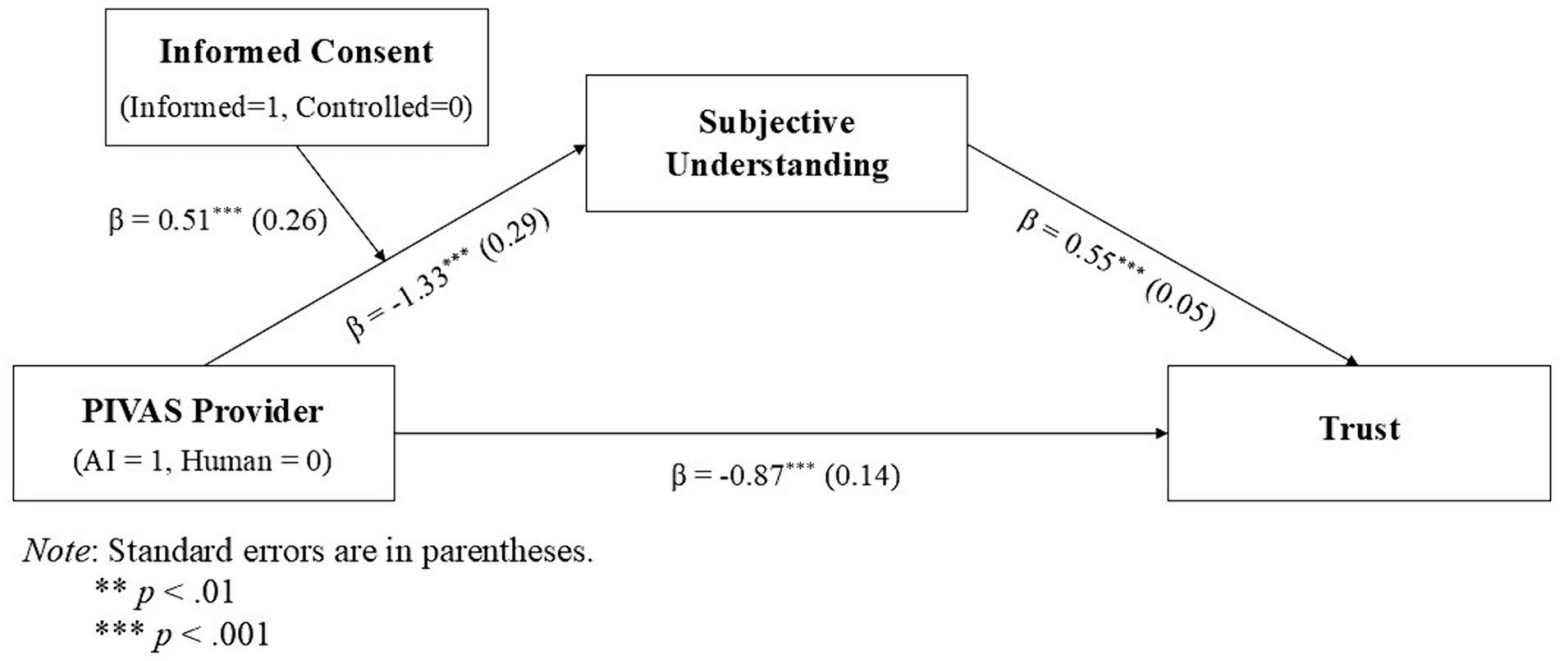
Direct and indirect effects of moderated mediation.

**Figure 6:**
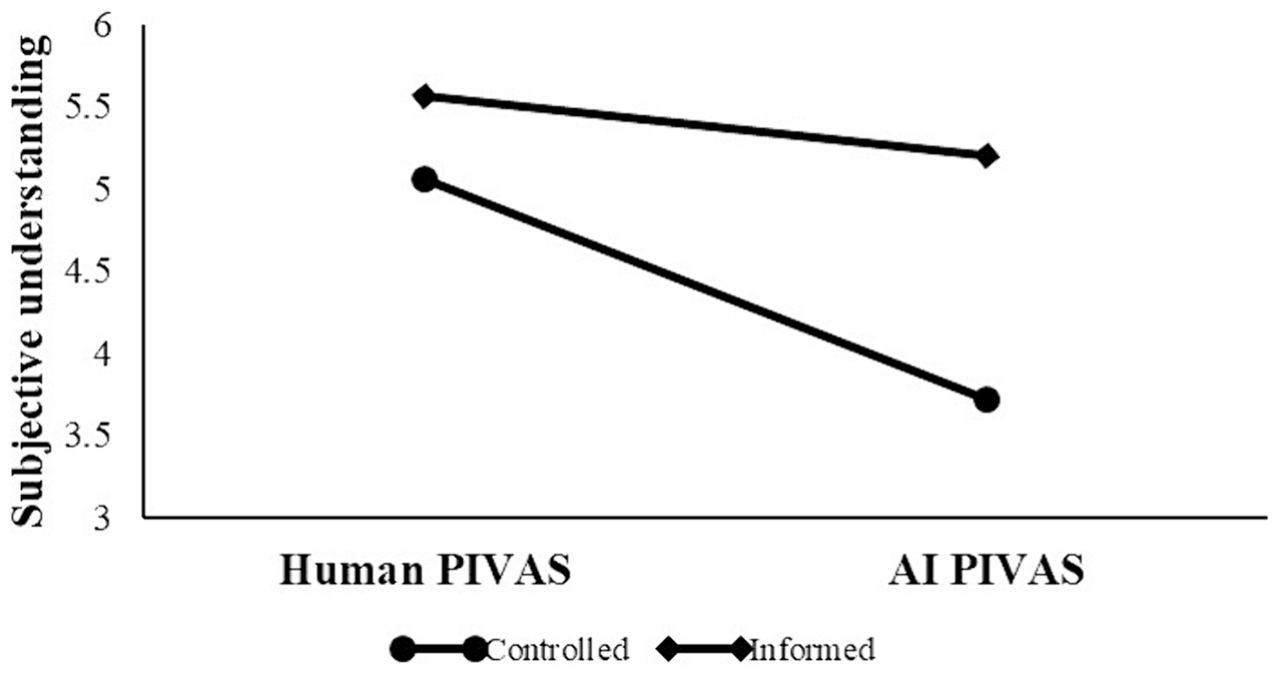
Simple slopes plot of the interaction.

A moderated mediation analysis using bootstrapping (PROCESS Model 7) with PIVAS provider as the independent variable (0 = human, 1 = AI), informed consent as the moderator (0 = controlled, 1 = informed), subjective understanding as the mediator, and trust as the dependent variable indicated a significant moderated mediation (index = 0.54, *SE* = 0.21, *CI*_95_ = [0.13, 0.95]), suggesting that the negative effect of AI PIVAS on patients’ trust, through subjective understanding, was stronger without informed consent (indirect effect = −0.74, *SE* = 0.19, *CI*_95_ = [−1.13, −0.39]) than with informed consent (indirect effect = −0.20; *SE* = 0.14; *CI*_95_ = [−0.49, 0.05]). Figure 5 shows the results from the direct and indirect effects of PIVAS provider on patients’ trust through the interaction (moderated mediation) with informed consent (also see Figure 6 with a simple slopes plot of the conditional effect of this interaction).

### Discussion

Study 5 demonstrated that informed consent significantly moderated the effect of AI PIVAS on subjective understanding. Patients’ subjective understanding of AI PIVAS was significantly improved by informed consent, resulting in increased trust, while patients’ subjective understanding of human PIVAS did not change significantly. Study 5 provided an effective medical practice to address the issue of trust in AI PIVAS that is the use of informed consent to make AI PIVAS explainable and thereby to improve patients’ subjective understanding of AI. Given the popularity of AI PIVAS, the findings of Study 5 will provide meaningful guidance for medical practice.

## General discussion

In five studies, we observed a lack of trust in AI PIVAS. In addition, we clarified the mediating effect of subjective understanding and the role of illusion of understanding (vs. difficulty in understanding). Finally, we verified the moderating effect of informed consent. Specifically, in Study 1, we found patients were reluctant to use AI PIVAS than human PIVAS due to lower trust in AI robots, which was demonstrated again in Study 2. This is in line with recent findings (Xie et al., 2024; Castelo et al., 2023; Zhang et al., 2023). In Study 3, we provided evidence that patients’ reluctance to trust in AI PIVAS was due to their lack of subjective understanding. Study 4 further showed that the reason patients preferred to trust in human PIVAS was due to the illusion of understanding humans and the difficulty in understanding AI; that is, they subjectively believed they understood humans well, while in fact they understood neither AI PIVAS nor human PIVAS. In Study 5, we manipulated informed consent to demonstrate that it moderates the relationship between AI PIVAS and subjective understanding. Informed consent improved patients’ subjective understanding of AI PIVAS and thereby enhanced trust.

### Theoretical implications

Our study contributes to the literature related to algorithm aversion and the adoption of AI. It provides a new perspective and thus contributes to a better understanding of the phenomenon of resistance to AI in a high-risk context. Researchers have identified various factors that influence trust in AI (see Kaya et al., 2024; Jackson et al., 2023; Bergner et al., 2023; Huang and Wang, 2023; Ismatullaev and Kim, 2024; Choung et al., 2023; Clegg et al., 2024; Usman et al., 2024). In particular, transparency has been shown to be key to trusting new technologies (Li et al., 2024). We extend this line of research by showing that one of the causes of algorithm aversion is that people do not understand how algorithms work (Yeomans et al., 2019). The findings show that patients lack subjective understanding of AI compared to humans, which may be the reason why AI PIVAS is less trusted than human PIVAS. In addition, previous works has focused on algorithm aversion in the context of AI diagnosis, with limited attention in the context of AI therapy. AI therapy and AI diagnosis are two different medical procedures, and they have unique impact on patients. Thus, we contribute to the literature on AI therapy.

Moreover, the study contributes to the literature on cognitive psychology. People often overestimate their understanding of things, but such understanding is often illusory (Xu et al., 2024; Cadario et al., 2021). The illusion of explanatory depth has mostly been recorded in mechanical devices and natural phenomena (Bonezzi et al., 2022), and it has been represented as a superficial understanding of how something works. Our study in AI shows that the reason for the higher trust of human PIVAS and lower trust of AI PIVAS is the illusion of understanding humans and difficulty in understanding AI, which ultimately influence people’s trust in decisions. Therefore, we extend the application scenarios for the illusion of explanatory depth and extend their scope to human decision making.

### Managerial implications

Given the complexity of medical AI, companies tend to emphasize the benefits of algorithms, such as accuracy, convenience and speed, in their marketing processes, while providing few details about how the algorithms work. For example, a study of a skin cancer App found that 57–64% of the descriptions in the App were performance-related, while only 21% were process-related (Cadario et al., 2021). Thus, improving subjective understanding of how medical AI works can provide useful insights not only for improving adoption but also for hospitals seeking to improve healthcare services. Our study provides practical insights for reducing mistrust in medical AI. Through the provision of informed consent, healthcare institutions can open up the “black box” to patients and users. The use of informed consent to explain the criteria or process of healthcare increases the trust in algorithm-based AI PIVAS, and this approach can be easily adapted to other fields and procedures.

Notably, in addition to using informed consent as a form of textual explanation, healthcare institutions can adopt other types of explanations to improve patients’ understanding of medical AI, such as visual explanations (Subramanian et al., 2024). Therefore, healthcare institutions can expose medical AI more vividly in front of patients and subconsciously train them through photo exhibitions and animation production. Healthcare institutions can even just reassure patients that they can get explanations when they want, because believing that an explanation is available may foster an illusion of understanding AI even if patients have not read the explanation (Ostinelli et al., 2024).

For AI designers, we suggest that with Natural Language Generation technology, AI can dynamically generate concise and easy-to-understand health reports based on a patient’s health data and diagnostic results, or adjust the linguistic complexity of the explanations based on the patient’s background, health status, and comprehension ability. Additionally, natural language processing technology can be adopted, allowing patients to talk to the device via voice, ask questions and get instant explanations. This interactive design could help patients who are unfamiliar with the technology use medical AI medica more easily.

### Limitations and future research

Despite the meaningful results, there are certain limitations in this study. First, the experiments in our study used hypothetical medical scenarios in which participants had to imagine themselves seeking help because of the described disease and then assess their trust. Future research can replicate the results with lab experiment where participants consider these scenarios in more natural settings. Second, the age of samples was on average around 30 years old, and further sampling among an older population might influence the results. The importance of treatment may vary by age, especially in older groups. The older population may feel that the way they receive treatment is more important than the younger and middle-aged populations do. Future research could investigate a broader population to enhance the robustness of the results. Third, our study ignores an important context for AI services that is service failure. Indeed, users often lack an understanding of why AI systems fail (Chopra et al., 2024). Future research could explore how to provide a clear rationale for AI-driven choices and actions to mitigate the damaging effects of service failures on consumer confidence and trust.

This study suggests that the reason patients trust human PIVAS more than AI PIVAS is that they have an illusion of understanding humans and difficulty in understanding AI. Future research could explore other consequences associated with the illusion of understanding. Our study shows that an illusion of understanding humans can generate stronger trust in human PIVAS. However, it can also have the opposite effect. People may incorrectly project their own biases onto others rather than the algorithm and consequently trust humans less than algorithms (Bonezzi and Ostinelli, 2021). For example, people may think that job recruiters prefer men than women, because they hold an internal bias that women work at a higher cost than men (e.g., maternity leave). In this case, an illusion of understanding humans may instead generate greater trust in AI. On the other side, people may also have an illusion of understanding AI. In situations involving fairness, people believe that AI makes fairer decisions than humans do (Xu et al., 2024). This needs to be further tested by future research.

## Data Availability

The original contributions presented in the study are included in the article/Supplementary material, further inquiries can be directed to the corresponding author.
