# Supplementary material for: The effect of subjective understanding on patients’ trust in AI pharmacy intravenous admixture services
Source: Front Psychol. 2024 Sep 5;15:1437915. doi: 10.3389/fpsyg.2024.1437915 (PMC11412255; doi:10.3389/fpsyg.2024.1437915)
Supplement: Supplementary file 1 [file Presentation_1.pdf]

Appendix 1: Introductory stimuli used in Study 2-5

General introduction

Imagine that you have recently developed a fever and feel ill because you have a rhinovirus infection. You decide to go to a well-known hospital for treatment. After diagnosis, the doctor determines that your condition is more serious and that you need an infusion to recover better, and prescribes one. You follow the doctor's advice and are ready to receive the infusion (Study 2).

Imagine that you felt sick because of an infection caused by a fall and subsequent injury. You decide to go to a well-known hospital for treatment. After diagnosis, the doctor determines that your condition is more serious and that you need an infusion to recover better, and prescribes one. You follow the doctor's advice and are ready to receive the infusion (Study 3).

Imagine that you were suffering from diarrhea and feeling ill because they had eaten spoiled food. You decide to go to a well-known hospital for treatment. After diagnosis, the doctor determines that your condition is more serious and that you need an infusion to recover better, and prescribes one. You follow the doctor's advice and are ready to receive the infusion (Study 4).

Imagine that you felt sick because they had recently suffered from acute gastritis. You decide to go to a well-known hospital for treatment. After diagnosis, the doctor determines that your condition is more serious and that you need an infusion to recover better, and prescribes one. You follow the doctor's advice and are ready to receive the infusion (Study 5).

Introduction of PIVAS

[In the human PIVAS condition]: The hospital provides Pharmacy Intravenous Admixture Services that all medications are prepared by pharmacists.

[In the AI PIVAS condition]: The hospital provides Pharmacy Intravenous Admixture Services that all medications are prepared by AI robots.

Appendix 2: Stimulus pictures in Study 2-5

	Human PIVAS	AI PIVAS
Study 2	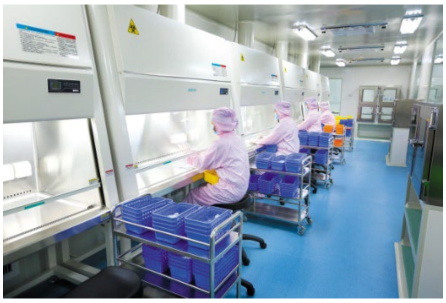	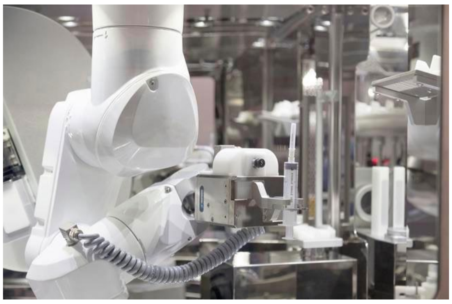
Study 3	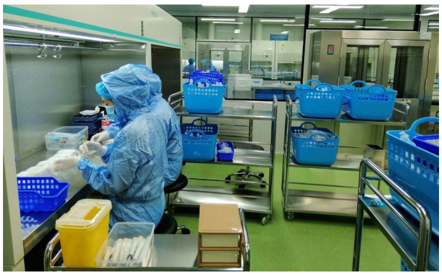	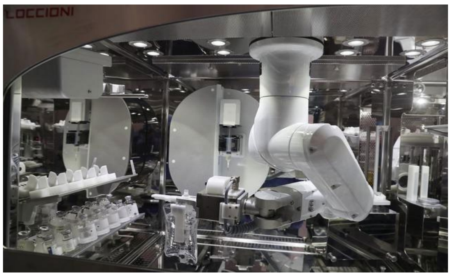
Study 4	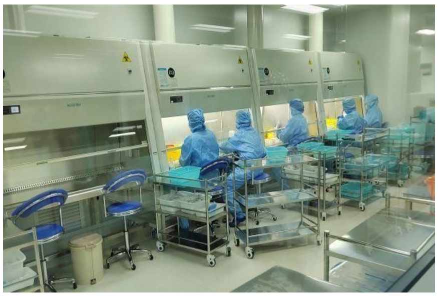	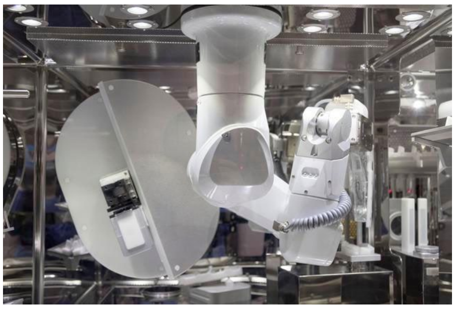
Study 5	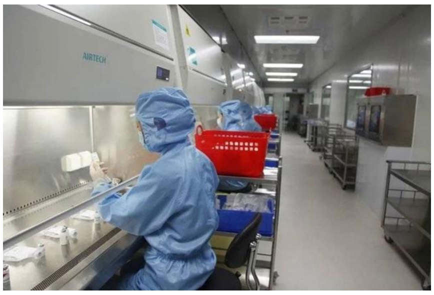	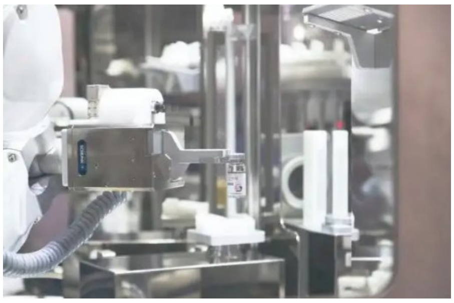

Source: All pictures are from the Internet

Appendix 3: Open-ended explanation in Study 3

Now, we would like to probe your knowledge a little bit more. We would like you to explain how you think a pharmacist (AI robot) prepares the medication in the PIVAS center.

Using the space below, please describe all the details you know about how a pharmacist (AI robot) prepares the medication, going from the first step to the last, and providing the causal connection between the steps.

In other words, please write about your knowledge of the following:

What are the steps that a pharmacist (AI robot) takes when preparing the medication? What do you know about the order of these steps? What do you know about the factors

that a pharmacist (AI robot) considers, and the factors that a pharmacist (AI robot) does not consider when preparing the medication?

Try to tell as complete a story as you can, inclusive of all the steps and the factors that you know a pharmacist (AI robot) considers.

Important: please do not look up or Baidu this topic online, as that would defeat the purpose of our survey -- we are interested in how people would explain how a pharmacist (AI robot) conducts these medical tests. So please just answer the following question to best of your knowledge.

As per your knowledge, how does a pharmacist (AI robot) prepare the medication in the PIVAS center?

[Text entry open-ended question with 50 characters minimum]

Appendix 4: Measures of objective understanding in Study 3

Objective Understanding:

How many times does xxx need to check prescription during the medication preparing.

- One time
- Two times
- Three times

Which of the following does xxx do in the process of preparing the medicine.

- Tilt and pull the syringe
- Pull the syringe vertically
- Pulling the syringe horizontally

What is the approximate residual rate of the medication prepared by xxx.

- 5%
- 1%
- 3%

Note: xxx represents a pharmacist or an AI robot.

Appendix 5: Manipulation of informed consent in Study 5

XXX Hospital Outpatient General Informed Consent Form

Dear patient:

Welcome to the xxx hospital for treatment. We will do our best to provide you with good service under the existing conditions. The hospital provides pharmacy intravenous admixture services (PIVAS) for patients that all medications are prepared by pharmacists (AI robots). We would like to inform you of the following matters and hope you will understand and cooperate with us.

1. I voluntarily seek medical help at xxx hospital and receive intravenous therapy as deemed necessary by the medical professional provided by the physicians (AI robots). I sign this consent form for the scope of my medical treatment, which refers to the outpatient and intravenous therapy received at the xxx hospital. I may ask the physicians (AI robots) of xxx hospital any questions about the treatment and medication I am receiving at any time.

2. According to the relevant regulations, the hospital will strictly refer to the national code of practice on PIVAS, Including: *Receiving prescriptions → Reviewing prescriptions → Printing infusion labels → Arranging and verifying medication → Adding medication and mixing it → Verifying and packaging finished infusions → Distributing and delivering → Checking and signing for receipt in the ward (AI robots receive medical advice → Pharmacists review medical advice → Pharmacists print QR code label → Pharmacists arrange and verify medication → Robots scan QR code → Pharmacists place medication → Robots check prescription information and identifies medication → Robots automatically mix and blend → Removing infusion bag → Checking medication → Signing → Passing out of warehouse).*

3. I understand and am willing to bear the cost of services charged by xxx Hospital, including non-essential medical treatment not covered by insurance.

If you understand the rules regarding charging by PIVAS, please sign to show your awareness.

Please sign here:

Note: texts in brackets are for the AI PIVAS conditions, and otherwise for the human PIVAS conditions.
